# Prognostic Significance of *ESR1* Amplification and *ESR1* PvuII, CYP2C19*2, UGT2B15*2 Polymorphisms in Breast Cancer Patients

**DOI:** 10.1371/journal.pone.0072219

**Published:** 2013-08-08

**Authors:** Aleksandra Markiewicz, Marzena Wełnicka-Jaśkiewicz, Jarosław Skokowski, Janusz Jaśkiewicz, Jolanta Szade, Jacek Jassem, Anna J. Żaczek

**Affiliations:** 1 Department of Medical Biotechnology, Intercollegiate Faculty of Biotechnology, University of Gdańsk and Medical University of Gdańsk, Gdańsk, Poland; 2 PostgraduateSchool of Molecular Medicine, Medical University of Warsaw, Warsaw, Poland; 3 Department of Oncology and Radiotherapy, Medical University of Gdańsk, Gdańsk, Poland; 4 Bank of Frozen Tissues and Genetic Specimens, Department of Medical Laboratory Diagnostics, Medical University of Gdańsk, Gdańsk, Poland; 5 Department of Surgical Oncology, Medical University of Gdańsk, Gdańsk, Poland; 6 Department of Pathology, Medical University of Gdańsk, Gdańsk, Poland; IPO, Inst Port Oncology, Portugal

## Abstract

**Introduction:**

Amplification of the *ESR1* gene, coding for estrogen receptor alpha, was shown to predict responsiveness to tamoxifen, however its prognostic impact in breast cancer patients has not been thoroughly investigated. Other factors that could contribute to responsiveness to tamoxifen treatment are polymorphisms in *ESR1* gene and genes involved in tamoxifen metabolism.

The aim of this study was to assess the prognostic role of *ESR1* gene dosage in a consecutive group of breast cancer patients and to correlate this feature with clinico-pathological factors. Additionally, *ESR1* PvuII, CYP2C19*2 and UGT2B15*2 polymorphisms were analyzed in the tamoxifen-treated subgroup of patients.

**Materials and Methods:**

Primary tumor samples from 281 stage I-III consecutive breast cancer patients were analyzed for *ESR1* gene dosage using real-time PCR with locked nucleic acids hydrolysis probes. In the tamoxifen-treated subgroup of patients, *ESR1* PvuII, CYP2C19*2 and UGT2B15*2 polymorphism in leukocytes genomic DNA were analyzed. Results were correlated with clinico-pathological factors and with disease-free survival (DFS) and overall survival (OS).

**Results:**

*ESR1* amplification (with a cut-off level of 2.0) was found in 12% of the entire group of breast cancer patients, and in 18% of the ER-negative subgroup. This feature was associated with decreased DFS both in the entire group (*P*=0.007) and in the ER-negative subgroup (*P*=0.03), but not in the tamoxifen-treated patients.

Patients with *ESR1* PvuII wt/wt genotype and at least one *UGT2B15* wt allele had a worse DFS (*P*=0.03) and showed a trend towards decreased Os (*P*=0.08) in comparison to patients with *ESR1* PvuII wt/vt or vt/vt genotype and *UGT2B15* *2/*2 genotype.

**Conclusions:**

*ESR1* amplification can occur in ER-negative tumors and may carry poor prognosis. In the tamoxifen-treated subgroup, poor prognosis was related to the combined presence of *ESR1* PvuII wt/wt and *UGT2B15*wt/wt or wt/*2 genotype.

## Introduction

Estrogen receptor (ER) facilitates normal development of mammary gland [1] but it is also involved in stimulating the growth of ER-positive breast cancers [2]. In the clinic of breast cancer, ER is a well-established predictive factor for the efficacy of endocrine therapies [3]. ER-positivity indicates the dependence of the tumor on ER-related pathways for survival and sustained growth [4] and increased gene dosage of estrogen receptor alpha gene (*ESR1*), may influence the function of complex internal ER signaling pathways in breast cancer cells. The frequency of *ESR1* gene amplification varies largely from 1% [5] to 23% [6] depending on the method of *ESR1* amplification analysis. Clinical significance of this abnormality is unclear as it was found to predict both endocrine therapy responsiveness [6,7] and resistance [8]. Since most studies on *ESR1* amplification included tamoxifen-treated groups [6–9], prognostic significance of *ESR1* gene amplification in general population of breast cancer patients is unclear.

Apart from *ESR1* amplification, polymorphisms in genes metabolizing tamoxifen or polymorphisms in *ESR1* gene can influence the response to tamoxifen. Polymorphism in the intron 1 of the *ESR1* gene (rs2234693), also called *ESR1* PvuII, was associated with increased risk of breast cancer [10] and decreased ER expression [11]. CYP2C19 is an enzyme converting tamoxifen to its active metabolites [12] and certain polymorphisms in this gene, including CYP2C19*2, were shown to be associated with decreased enzymatic activity leading to poor metabolizer phenotype (*2/*2) [13]. Polymorphism in the enzyme UDP glucuronosyltransferase 2 family, polypeptide B15 (rs1902023 also named UGT2B15*2) participating in glucuronidation of tamoxifen metabolites [14] was associated with its decreased enzymatic activity and slower glucuronidation and secretion of active tamoxifen metabolites [15]. All these polymorphisms were also associated with survival of breast cancer patients treated with endocrine therapies, but the data are limited and inconclusive. Whereas *ESR1* PvuII polymorphism correlated with decreased survival [16], increased survival for UGT2B15 *2 allele [16] and CYP2C19 *2 allele [17] patients as well as decreased survival for UGT2B15 *2/*2 [18] and CYP2C19 *2/*2 [16] patients was observed. 

This study aimed at assessing the prognostic role of *ESR1* gene dosage in consecutive group of breast cancer patients and to correlate *ESR1* gene dosage with their clinico-pathological data. Additionally, prognostic value of *ESR1* PvuII, CYP2C19*2 and UGT2B15*2 polymorphism was analyzed in a tamoxifen-treated subgroup of patients.

## Patients and Methods

### Ethics Statement

The study was conducted in accordance with the Helsinki Declaration and accepted by the Ethics Committee of the coordinating center, the Medical University of Gdansk (NKEBN 16/2010, 781/2005). All patients signed informed consent forms.

### Clinical material

The study group included 281 consecutive stage **I**-**III** breast cancer patients (Table 1) treated between 1999 and 2009 in three Polish institutions. This group included both ER-negative and ER-positive patients. Primary tumor samples were obtained by surgical excision or excisional biopsy prior to any systemic treatment, snap-frozen in liquid nitrogen and stored at -80° C for further DNA isolation. Adjuvant treatment was offered according to the standards of care at the time of their diagnosis. Twenty-seven percent (75/281) of the patients were treated with tamoxifen 20 mg/day, either alone (46%) or in combination with radiotherapy (12%), chemotherapy (17%) or both (25%).

**Table 1:** Patients characteristics.

**Variable**	**Number of cases (%)**	
**Age**		
<50 yr.	68	(24)
≥50 yr.	213	(76)
**T stage**		
T1	91	(32)
T2	128	(46)
T3	28	(10)
T4	31	(11)
Missing data	3	(1)
**N stage**		
N0	148	(53)
N1	73	(26)
N2	50	(18)
N3	6	(2)
Missing data	4	(1)
**Grade**		
G1	14	(5)
G2	107	(38)
G3	85	(30)
Missing data	75	(27)
**HER2 status**		
Negative	182	(65)
Positive	29	(10)
Missing data	70	(25)
**ER status**		
Negative	120	(43)
Positive	161	(57)
**PgR status**		
Negative	131	(47)
Positive	150	(53)
**Histological type**		
Ductal	191	(68)
Lobular	51	(18)
Other	33	(12)
Missing data	6	(2)
**Molecular subtype**		
Luminal A	60	(21)
Luminal B (HER2-)	38	(14)
Luminal B (HER2+)	15	(5)
HER2+	14	(5)
Triple negative	56	(20)
Missing data	98	(35)
**TAM-treatment**		
Not-treated	206	(73)
Treated	75	(27)

Median age of the patients was 56 years (range 27-86 years, average 57.4 years). Follow-up data were available for 277 patients, median follow-up time was 54 months (range 0.2-137 months) and the dataset was frozen in August 2011. Seventy-three patients (26%) developed tumor recurrence and 41 (15%) have died. In accordance with St Gallen guidelines [19], tumors were divided into five surrogate intrinsic subtypes based on the expression of ER, PgR, HER2, tumor grade and/or Ki-67: 1) Luminal A–ER+ and/or PgR+, HER2-, Ki-67 below 14% or G1/2; 2) luminal B (HER2-negative) – ER+ and/or PgR+, HER2-, Ki-67 above 14% or G3; 3) luminal B (HER2-positive) – ER+ and/or PgR+, HER2+, any Ki-67 or any G; 4) HER2+ -ER- and PgR-, HER2+; 5) Triple negative (TNBC) – ER-, PgR-, HER2-. Details of procedures of ER, PgR, Ki-67 and HER2 status determination are presented in the Text **S1**.

In the tamoxifen-treated group, full blood samples were available from 69 patients. Samples were stored at -80° C and 200 µl was used for genomic DNA isolation with QIAamp DNA Blood Mini Kit (Qiagen, Germany) in accordance with the manufacturer’s instructions. From the macrodissected tumor samples, DNA was isolated using QIAamp DNA Mini Kit (Qiagen, Germany) in accordance with the Tissue protocol. Quantity and quality of the isolated DNA were measured on Spectrophotometer ND-1000 (NanoDrop, USA). Isolated DNA samples were stored at -20° C until the analyzes of *ESR1* gene dosage and *ESR1* PvuII, CYP2C19*2 and UGT2B15*2 polymorphisms.

Full blood samples were also drawn from 5 healthy donors (who signed informed consent forms) in order to isolate leukocyte DNA that served as a reference sample and an inter-run calibrator in real-time PCR experiments. Additionally, pooled genomic leukocytes DNA purchased from Roche was tested. Finally, we preserved healthy breast tissue samples obtained from 3 patients during surgery from a site distant from the primary tumor location. DNA from *ESR1*-amplified sarcoma cell line [5] was used as a positive control.

### *ESR1* Gene Dosage Evaluation

*ESR1* gene dosage was measured by real-time PCR on iCycler iQ thermal cycler (Bio-Rad, USA). Pfaffl relative quantification method was used for the calculations as described before [20], with modifications (inclusion of inter-run calibrator) applied by qbase Plus software version 2 [21]. Briefly, the amount of *ESR1* gene was related to the amount of amyloid precursor protein (APP) reference gene in a test sample and in a reference sample (leukocyte DNA) using gene specific amplification efficiencies. PCR was performed using following primers sets for *ESR1* gene (NC_000006.11) F 5’-ACA TGG ACA CCT CCC AGT C-3’, R 5’-ACA GAC TAA CAC AGC CCA TC-3’; and *APP* gene (NC_000021.8) F 5’-AGC CCA GAA GGT GTC AAA CA-3’, R 5’-CAT CTT CAT GTC CGT TGC AT-3’) and locked nucleic acid hydrolysis probe nr 4 and 2 for *ESR1* and *APP* detection (Universal ProbeLibrary Probes, Roche), respectively. The length of each amplicon was 60 bp.

The composition of reagents used in real-time PCR was as follows: 1× reaction buffer containing MgCl_2_, dNTPs and Taq polymerase (FastStart Taqman Probe Master (no ROX), Roche), primers 600 nM of each, probe 250 nM. One hundred ng of DNA, in the volume of 5 µl, and water were added into the reaction mixture to obtain the final volume of 20 µl. Reactions were performed in duplicates on 96-well plates (Bio-Rad, USA), sealed with optical tape (Bio-Rad, USA). On each plate negative control (no template DNA) for each gene and inter-run calibrator (DNA from leukocytes) were always included. Real-time PCR cycling conditions were 10 min 95° C, cycles 1-45: 15 s 95° C, 1 min 60° C. Specificity of the primers was checked by electrophoretic separation of post-PCR solution on 2% agarose gel stained with ethidium bromide.

### Polymorphisms analysis

Presence of three polymorphisms connected with tamoxifen metabolism - CYP2C19*2 (rs4244285, G>A), UGT2B15*2 (rs1902023 A>C) and *ESR1* PvuII (rs2234693 T>C) was tested. Polymorphisms in *ESR1* and *CYP2C19* genes were analyzed with restriction fragment length polymorphism (RFLP) using PvuII and SmaI restriction enzymes, respectively. At first, genes were amplified in PCR using primer sequences described before for *ESR1* F 5’-CTG CCA CCC TAT CTG TAT CTT TTC CTA TTC TCC-3’, R 5’-TCT TTC TCT GCC ACC CTG GCG TCG ATT ATC TGA-3’ [22] and *CYP2C19* F 5’-AAT TAC AAC CAG AGC TTG GC-3’, R 5’-TAT CAC TTT CCA TAA AAG CAA G-3’ [23]. PCR reactions were performed with following concentrations: 300 nM of each primer, 200 µM of dNTPs, 2 mM (for *CYP2C19*) or 4 mM (for *ESR1*) of MgCl_2_ and 1U of Taq polymerase (Promega, USA). One hundred ng (for *ESR1*) or 150 ng (*CYP2C19*) of DNA was added per reaction and the total reaction volume was 25 µl. Amplification conditions were described before [24] and were as follows: for the *CYP2C19* 95° C for 2 min – initial denaturation, 35 cycles of denaturation at 94° C for 20 s, annealing 53° C for 30 s and elongation 72° C for 30s, final extension 72° C for 7 min; for *ESR1* gene 95° C for 2 min – initial denaturation, 35 cycles of denaturation at 94° C for 30 s, annealing 62° C for 30 s and elongation 72° C for 30 s, final extension 72° C for 7 min. Ten µl of post-PCR solutions was digested with 10U of restriction enzyme PvuII (for *ESR1* PvuII detection) or SmaI (for CYP2C19*2 detection) in 1xreaction buffer G or Tango, respectively. Final sample volume was 31 µl and the reaction was carried out in an incubator for 2 hours at 37° C (for PvuII) or in a thermal cycler (Mastergradient, Eppendorf) at 30° C (for SmaI). Enzymes were inactivated by the addition of EDTA pH 8 to a final concentration of 20 mM (for PvuII) or 20 min incubation at 65° C (for SmaI). Samples were then separated on 3% (*CYP2C19*) of 1.5% (*ESR1*) agarose gel in 1xTAE buffer at 115V for 40 min. Gels were stained with Gel Red (Gentaur, Belgium) and visualized under UV light in GelDoc System (Bio-Rad, USA). ESR1 amplicon had a length of 1.3 kbp and it was digested to fragments of 850 bp and 450 bp by PvuII enzyme if the wild-type (wt) T nucleotide was present in the enzyme recognition sequence. Mutated allele containing C nucleotide in the polymorphic locus (enzyme recognition sequence) was not digested by the PvuII enzyme. CYP2C19 amplicon had a length of 169 bp and it was digested by SmaI enzyme to achieve fragments of 120 bp and 49 bp if the wt allele G was present, mutated allele containing A in the recognition sequence was not digested.

Polymorphism in UGT2B15 gene was analyzed with high resolution melting (HRM) on CFX96 thermal cycler (Bio-Rad, USA) using Maxima SYBR Green qPCR Mastermix (Thermo Scientific, USA). Primers were designed using Primer3 software and had following sequences: F 5’-CAC CAT ATA TCC ATC TAT CGA GAA-3’ and R 5’-TCA ATG CCA GTA AAT CAT CTG C-3’. Reactions were performed in duplicates in a final volume of 20 µl adding 10 ng of DNA per reaction and 300 nM of each primer. Applied real-time PCR conditions were: 10 min at 95° C, 40 cycles of denaturation at 95° C for 15 s and annealing/extension at 60° C for 1 min. Melt curve analysis began from gradual temperature increase from 55°C to 94°C in 0.2°C increments and 10 s plate read. Post-HRM data were analyzed using Precision Melt Analysis Software version 1.0 (Bio-Rad, USA). Start and end fluorescence signal were automatically normalized. Each curve on a fluorescence difference plot was inspected visually in order to determine the genotype. Every plate contained two positive control samples (for each genotype) that were previously sequenced in order to determine nucleotide composition of the polymorphic locus.

### Statistical analysis

All statistical analyses were performed using the STATISTICA software, version 10. Disease-free survival (DFS) and overall survival (OS) were computed using Kaplan-Meier method and compared using log-rank test. DFS was defined as the time from surgery to an event (local or distant relapse, second malignancy or death, whichever came first) or censoring. Censoring was defined as lost to follow-up or survival without relapse at the end of follow-up. OS was defined as the time from surgery to death or censoring. Cox proportional hazards regression analysis was used to identify the independent predictors of DFS and OS. *P* values ≤0.05 were considered significant. The study was performed in accordance with the REMARK criteria [25].

In the survival analysis, patients were divided into two groups: *ESR1*-normal and *ESR1*-amplified applying a cut-off value of 2.0, as described by other groups [6,7,26]. Correlations between *ESR1* and other variables were performed using *ESR1* gene dosage as a continuous variable.

## Results

Efficiencies of *ESR1* and *APP* gene amplification were 1.91 and 1.93, respectively. Average *ESR1* gene dosage in the entire group of patients was 1.27±0.64 (median 1.11, range 0.12-4.74). Average *ESR1* gene dosage in the ER-negative and ER-positive subgroups was 1.36±0.80 (median 1.20, range 0.12-4.74) and 1.17±0.47 (median 1.10, range 0.24-2.66), respectively. With the cut-off level of 2.0, 12% (33/281) of the samples were classified as *ESR1*-amplified, in the ER-negative subgroup 18% (22/120). The average *ESR1* gene dosage in healthy breast tissues was 0.94±0.20, in pooled genomic DNA of healthy donors 1±0.05 and in *ESR1*-amplified sarcoma cell line 5.37±0.25.

### Correlation of *ESR1* gene dosage with clinical and pathological data

*ESR1* gene dosage in the entire group of patients correlated with higher T stage (*P*=0.01, Table **2**) and lymph node involvement (*P*=0.008, Table **2**), whereas in the tamoxifen-treated group the only correlation included younger age (*P*=0.05) (Table **2**). There was no correlation between *ESR1* gene dosage and ER-status or surrogate intrinsic molecular breast cancer subtypes (Table **2** and Figure **S1**).

**Table 2:** Correlation between *ESR1* gene dosage and clinico-pathological data in the entire group and in the tamoxifen-treated patients.

**Variable**	**All patients**			**Tamoxifen-treated patients**		
	**N**	**Median *ESR1* gene dosage (25-75 percentile)**	***P***	**N**	**Median *ESR1* gene dosage (25-75 percentile)**	***P***
**T stage**						
T1-2	219	1.11 (0.85-1.39)	**0.01**	67	1.10 (0.86-1.24)	0.85
T3-4	59	1.25 (0.88-1.97)		7	1.13 (0.63-1.18)	
**N stage**						
0	148	1.08 (0.82-1.31)	**0.008**	54	1.11 (0.85-1.24)	0.78
1	129	1.17 (0.91-1.65)		19	1.07 (0.85-1.17)	
**Grade**						
1-2	121	1.11 90.89-1.41)	0.87	29	1.08 (0.85-1.17)	0.54
3	85	1.16 (0.87-1.47)		15	1.10 (0.92-1.21)	
**Histological type**						
Ductal	191	1.13 (0.87-1.56)	0.12	42	1.10 (0.91-1.23)	0.45
Lobular	51	1.01 (0.82-1.31)		19	0.95 (0.74-1.24)	
**ER status**						
Negative	120	1.20 (0.86-1.64)	0.12	22	1.10 (0.90-1.49)	0.26
Positive	161	1.10 (0.85-1.37)		53	1.08 (0.84-1.20)	
**PR status**						
Negative	131	1.11 (0.83-1.65)	0.75	22	1.10 (0.84-1.25)	1
Positive	150	1.11 (0.88-1.39)		53	1.10 (0.85-1.23)	
**HER2 status**						
Negative	182	1.10 (0.83-1.37)	0.75	47	1.08 (0.83-1.21)	0.50
Positive	29	1.11 (0.90-1.31)		9	1.20 (0.88-1.23)	
**Age**						
<50 yr.	68	1.18 (0.88-1.51)	0.53	19	1.20 (0.99-1.55)	**0.05**
≥50 yr.	213	1.10 (0.85-1.47)		56	1.06 (0.80-1.17)	
**Molecular subtype**						
Luminal A	60	1.10 (0.85-1.39)	1	13	1.08 (0.85-1.11)	0.71
Luminal B (HER2-)	38	1.13 (0.40-2.07)		11	1.05 (0.83-1.21)	
Luminal B (HER2+)	15	1.17 (0.92-1.27)		8	1.21 (0.90-1.27)	
HER2+	14	1.02 (0.87-1.71)		1	0.87	
Triple negative	56	1.07 (0.80-1.43)		5	1.13 (0.63-1.16)	
**Allred score**						
0-2	20	1.03 (0.88-1.29)	0.56		-	
3-6	27	1.12 (0.87-1.41)				
7-8	41	1.18 (0.90-1.58)				
***ESR1* PvuII**						
wt/wt		Not analyzed		22	1.19 (1.10-1.31)	**0.0003**
wt/vt				30	1.07 (0.85-1.17)	
vt/vt				16	0.77 (0.64-1.03)	
***CYP2C19***						
wt/wt		Not analyzed		56	1.10 (0.84-1.24)	0.58
wt/*2				10	1.11 (0.97-1.18)	
*2/*2				2	0.89 (0.72-1.05)	
***UGT2B15***						
wt/wt		Not analyzed		19	1.04 (0.73-1.18)	0.54
wt/*2				33	1.10 (0.90-1.20)	
*2/*2				15	1.11 (0.88-1.31)	

### Correlation of ESR1 gene dosage with survival

**Figure 1:**
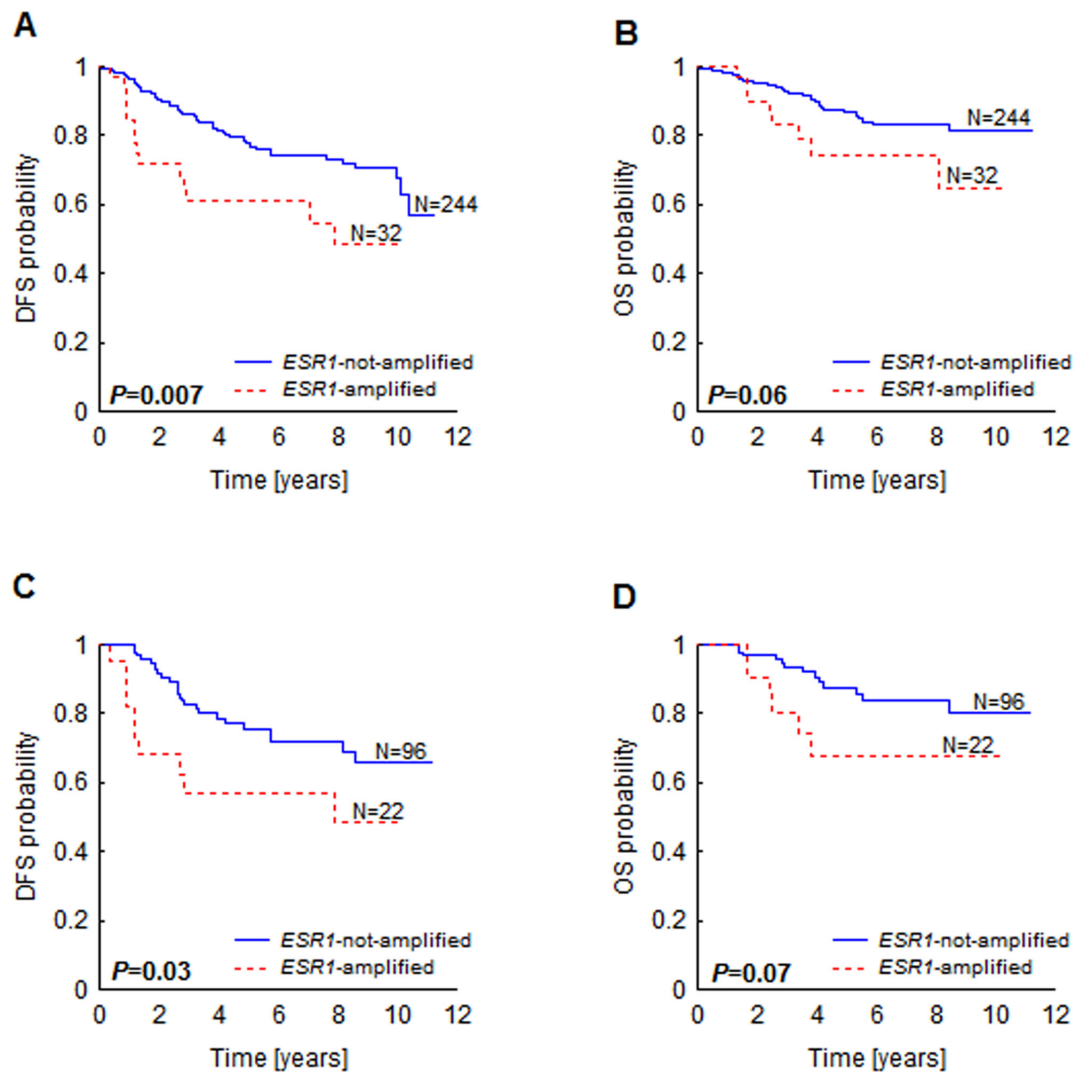
Kaplan-Meier survival curves according to *ESR1* gene amplification status. Probability of disease-free survival (A, C) and overall survival (B, D) in all patients (A, B) and in ER-negative patients (C, D).

In the entire group of patients (N=276), *ESR1* amplification (defined as *ESR1* gene dosage above 2) was associated with decreased DFS (*P*=0.007, Figure **1A**) and OS (only a trend, *P*=0.06, Figure **1B**). Similar results were obtained in the ER-negative patients (N=118) (DFS *P*=0.03, Figure **1C**, OS *P*=0.07, Figure **1D**); in the tamoxifen-treated subgroup (N=72) *ESR1* amplification showed no association with survival.

In the univariate analysis, *ESR1* gene dosage showed to be a poor prognostic factor for DFS and OS in the whole cohort of patients (DFS: HR 1.68, CI 1.25-2.26; OS:HR 1.61, CI 1.07-2.41) (Table **S1**). No association was found in the tamoxifen-treated subgroup of patients.

*ESR1* gen dosage did not sustain its prognostic value in the multivariate analysis (Table **S1**).

### *ESR1* PvuII, CYP2C19*2 and UGT2B15*2 polymorphisms

Frequencies of the polymorphic (variant) alleles were 46%, 10% and 46% for *ESR1* PvuII (rs2234693), CYP2C19*2 (rs4244285) and UGT2B15*2 (rs1902023), respectively. The details of expected versus observed genotypes frequencies are presented in Table 3. Genotypes distribution did not show significant deviation from the Hardy-Weinberg equilibrium as assessed by χ^2^ test.

**Table 3:** Number of cases (N) and percentages of observed and expected genotypes of *ESR1* PvuII, *CYP2C19* and *UGT2B15* in tamoxifen-treated patients.

	**N observed**	**% observed**	**N expected**	**% expected**	***P***
***ESR1* PvuII**					
wt/wt	22	32	20.4	29.5	0.43
wt/vt	31	45	34.2	49.6	
vt/vt	16	23	14.4	20.8	
Total	69	100	69	100	
***CYP2C19***					
wt/wt	57	83	55.7	80.7	0.09
wt/*2	10	14	12.6	18.2	
*2/*2	2	3	0.7	1	
Total	69	100	69	100	
***UGT2B15***					
wt/wt	20	29	19.8	28.8	0.94
wt/*2	34	49	34.4	49.7	
*2/*2	15	22	14.8	21.5	
Total	69	100	69	100	

**Figure 2:**
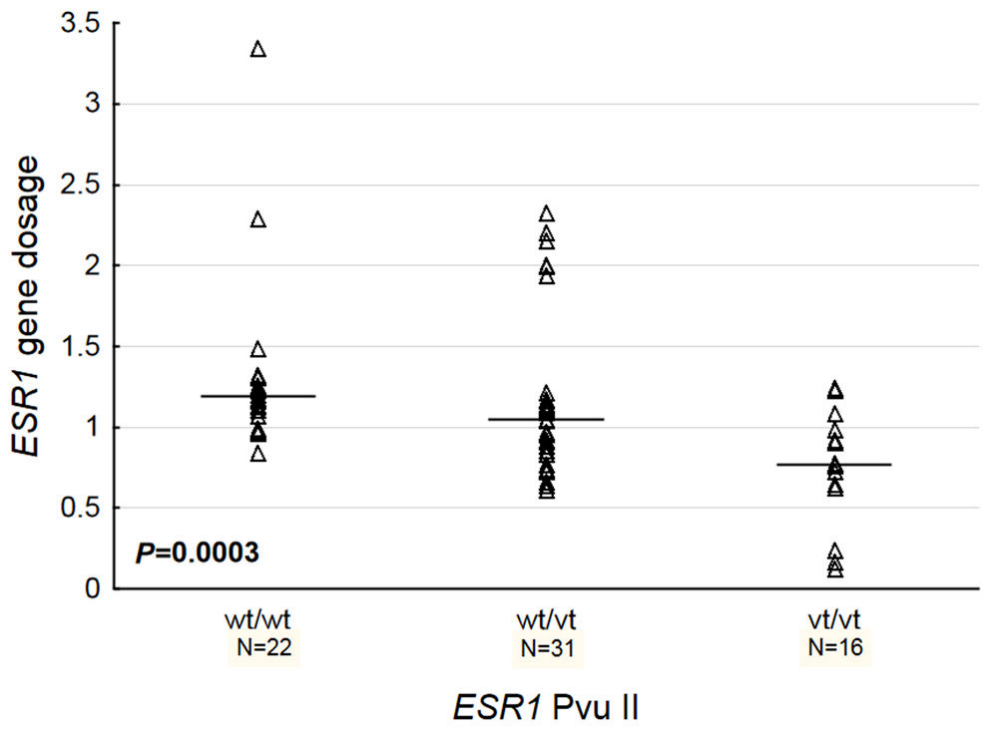
Correlation between *ESR1* gene dosage and *ESR1* PvuII polymorphism.

The presence of the *ESR1* PvuII polymorphism correlated with decreased *ESR1* gene dosage (*P*=0.0003, Figure **2** and Table **2**) and tended to correlate with ER-negativity (*P*=0.07, Table **4**). Moreover, the lack of both *ESR1* PvuII polymorphism and UGT2B15*2 polymorphism (analyzed separately) showed a trend towards decreased DFS (*P*=0.02 for *ESR1* PvuII and *P*=0.12 for *UGT2B15*) and OS (*P*=0.17 for *ESR1* PvuII and 0.32 for *UGT2B15*) (Table **4**). Combined status of *ESR1* PvuII and UGT2B15*2 polymorphism had stronger prognostic impact than each of the polymorphisms assessed alone.

**Table 4:** Correlation between *ESR1*, *CYP2C19, UGT2B15* polymorphisms and clinicopathological features.

	***ESR1* PvuII**			***CYP2C19***			***UGT2B15***		
	**wt/vt vt/vt**	**wt/wt**	***P***	**wt/wt**	wt/*2 *2/*2	***P***	**wt/wt**	wt/*2 *2/*2	***P***
**T stage**									
T1-2	43	18	0.15	51	10	0.68	15	46	0.09
T3-4	3	4		6	1		4	3	
**N stage**									
N0	34	16	0.51	41	9	0.43	13	37	0.51
N1	11	6		15	2		5	12	
**Grading**									
G1-2	20	6	0.31	21	5	0.59	6	20	0.31
G3	9	5		11	3		5	9	
**Histological type**									
Ductal	30	11	0.49	32	9	0.04	14	28	0.27
Lobular	11	5		16	0		3	12	
**ER status**									
0	12	6	0.55	15	3	0.62	7	11	0.22
1	35	16		42	9		13	38	
**PR status**									
0	9	11	0.01	18	2	0.25	7	14	0.40
1	38	11		39	10		13	35	
**HER2 status**									
0	31	12	0.54	36	7	0.49	14	28	0.34
1	7	2		7	2		2	8	
**Age**									
<50 yr.	10	6	0.40	13	3	0.57	4	11	0.55
≥50 yr.	37	16		44	9		16	38	
**Molecular subtype**									
Luminal A	10	2	0.72	9	3	0.73	3	9	0.31
Luminal B (HER2-)	9	2		10	1		3	8	
Luminal B (HER2+)	7	2		7	2		2	7	
HER2+	0	0		0	0		0	1	
Triple negative	2	2		4	0		3	1	
**Recurrence**									
Yes	40	13	0.02	43	10	0.43	13	40	0.12
No	7	9		14	2		7	9	
**Death**									
Yes	42	17	0.17	49	10	0.56	16	43	0.32
No	5	5		8	2		4	6	

**Figure 3:**
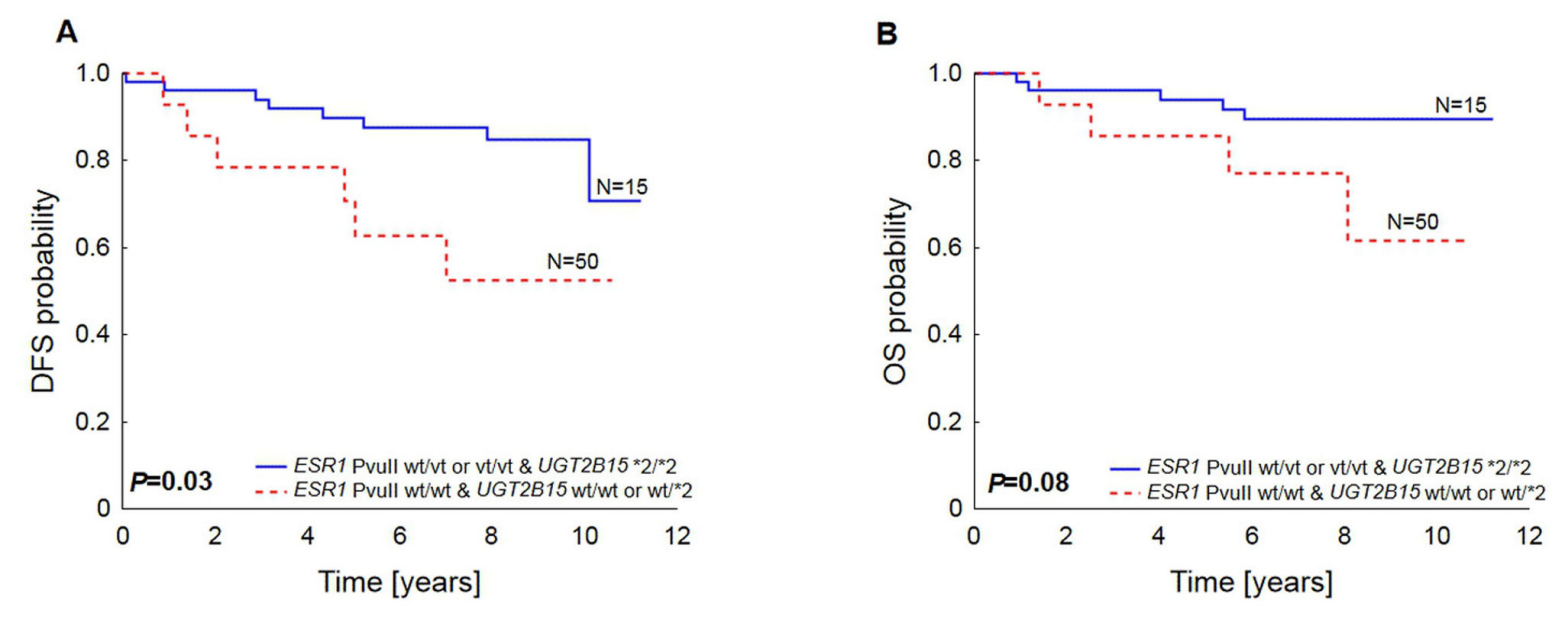
Patients survival in relation to *ESR1* PvuII and UGT2B15*2 polymorphisms in tamoxifen-treated patients. Disease-free survival (A) and overall survival (B) are shown in respective panels.

Patients with *ESR1* PvuII genotype wt/wt and with *UGT2B15* wt/wt or wt/*2 genotype had a worse DFS (*P*=0.03) and showed a trend towards decreased OS (*P*=0.08) in comparison to patients with *ESR1* PvuII wt/vt or vt/vt genotype and *UGT2B15* *2/*2 genotype (Figure **3**).

## Discussion

The issue of *ESR1* gene amplification in breast cancer has recently gained a lot of interest [5,7,26–29]. In the study from 2011, Nielsen et al. [8] hypothesized that “copy number changes of the *ESR1* gene confer resistance to tamoxifen because amplification is an abnormal status and normal ER protein expression (ER positive status) is requisite for response to tamoxifen“. Their study included only tamoxifen-treated patients for whom amplification of *ESR1* gene indeed conferred poor prognosis. We have extended this hypothesis assuming that *ESR1* amplification, as an abnormal state, could be a negative prognostic factor in a general population of breast cancer patients as it affects the natural course of breast cancer progression. Tumors that have amplification of *ESR1* rely more on endogenous estrogen and thus estrogen-driven mitogenic effect could be manifested in general population of breast cancer patients, independently of tamoxifen treatment.

Amplification of the *ESR1* gene was assessed in a number of studies using a variety of techniques, including real-time PCR, multiplex ligation-dependent probe amplification, fluorescent in situ hybridization (FISH), chromogenic in situ hybridization (CISH) and comparative genomic hybridization (CGH). To the best of our knowledge, we have used for the first time real-time PCR with locked nucleic acids (LNA) hydrolysis probes for *ESR1* gene dosage analysis. This approach combines advantages of real-time PCR method, such as high resolution, cost-effectiveness as well as higher sensitivity with increased specificity of LNA probes [30]. Moreover, in a recent overview of data from a number of publications studying *ESR1* gene dosage in breast cancer, PCR was shown to give the most coherent results across different studies [31]. Despite several studies, the significance of *ESR1* as a prognostic and predictive function remains unclear. In the study of Holst et al. [7] *ESR1* amplification was associated with longer OS of tamoxifen-treated patients; and Tomita et al. [6] showed longer DFS in a consecutive group of breast cancer patients. In contrast, in the study of Nielsen [8] including tamoxifen-treated patients, OS and DFS of breast cancer patients with *ESR1* amplification were decreased. Similar results were also reported by Lin et al. in a consecutive series of breast cancer patients [32]. *ESR1* amplification/gain was also not uniformly correlated with increased expression of ER protein [26] and was also found in ER-negative breast cancer patients [5,33], currently confirmed in our study.

We have found *ESR1* amplifications (*ESR1* gene dosage above 2.0) in 12% of the samples, and the association of this abnormality with T stage, lymph node involvement and decreased DFS and OS. We have not found such correlations in the tamoxifen-treated patients, therefore we sought for other factors that could affect tamoxifen-responsiveness. To this end, we have analyzed the occurrence of polymorphisms in three genes (*ESR1, CYP2C19* and *UGT2B15*) that were earlier shown to have prognostic significance in tamoxifen-treated breast cancer patients [16]. In our study, the presence of at least one *UGT2B15* wt allele combined with the wt/wt *ESR1* PvuII genotype was associated at a borderline level with decreased DFS and OS. *ESR1* PvuII wt allele also correlated with increased *ESR1* gene dosage and negative status of PgR protein. Poor prognosis of patients with *ESR1* PvuII wt/wt and *UGT2B15* wt/wt or wt/*2 genotype could be a result of insufficient inhibition of ER receptors by tamoxifen due to increased level of ER-protein and decreased level of active tamoxifen metabolites that are eliminated from the system at higher rates due to increased activity of UGT2B15 wt allele (A). A trend toward increased recurrence was also observed in tamoxifen-treated breast cancer patients carrying *UGT2B15*A allele [18] and in patients with *ESR1* PvuII vt allele [34]. No prognostic impact of CYP2C19*2 polymorphism in tamoxifen-treated breast cancer patients was also reported by other groups [35,36]. *ESR1* PvuII, CYP2C19*2 and UGT2B15*2 alleles frequencies observed in our study were within the ranges reported for European population -31-51%, 7-22% and 49-54%, respectively [37].

We are aware of several limitations of this study, including its retrospective nature and relatively small sample size. Especially the lack of prognostic value of *ESR1* gene dosage in tamoxifen-treated patients cannot be interpreted as a definitive result, as in this group the recurrence was still almost 2.5-times more frequent in the *ESR1*-amplified in comparison to *ESR1*-normal group of patients (29% vs. 12%). With the observed survival rates of patients and assuming an alpha of 5% and 80% power, a replication study with a minimal sample size of 128 would be required to validate our results concerning the impact of *ESR1* amplification on patients’ overall survival.

In summary, we have showed that *ESR1* amplification measured by real-time PCR with LNA probes is a poor prognostic factor in unselected breast cancer patients. In the tamoxifen-treated subgroup poor prognosis was related to the combined presence of *ESR1* PvuII wt/wt and *UGT2B15* wt/wt or wt/*2 genotype. We hope that further validation of these results and an attempt to understand basic biological changes driven by increased *ESR1* gene dosage as well as by *ESR1* PvuII and UGT2B15*2 polymorphisms will contribute to the understanding of breast cancer biology.

## Supporting Information

Text S1 Description of ER, PgR, Ki-67, HER2 status determination in formalin fixed paraffin embedded tumor samples.
(PDF)

Figure S1 ***ESR1* gene dosage in breast cancer samples according to ER status measured by IHC**.
(PDF)

Table S1 Univariate and multivariate analysis for DFS and OS according to molecular markers and clinicopathological variables.
(PDF)

## References

[1] Bocchinfuso WP, Korach KS (1997) Mammary Gland Development and Tumorigenesis in Estrogen Receptor Knockout Mice. J Mammary Gland Biol Neoplasia 2: 323-334. doi:10.1023/A:1026339111278. PubMed: 10935020. 10935020 10.1023/a:1026339111278

[2] Yoshidome K, Shibata MA, Couldrey C, Korach KS, Green JE (2000) Estrogen promotes mammary tumor development in C3(1)/SV40 large T-antigen transgenic mice: paradoxical loss of estrogen receptoralpha expression during tumor progression. Cancer Res 60: 6901-6910. PubMed: 11156389. 11156389

[3] EBCTCG (2005) Effects of chemotherapy and hormonal therapy for early breast cancer on recurrence and 15-year survival: an overview of the randomised trials. Lancet 365: 1687-1717. doi:10.1016/S0140-6736(05)66544-0. PubMed: 15894097. 15894097 10.1016/S0140-6736(05)66544-0

[4] Musgrove EA, Sutherland RL (2009) Biological determinants of endocrine resistance in breast cancer. Nat Rev Cancer 9: 631-643. doi:10.1038/nrc2713. PubMed: 19701242. 19701242 10.1038/nrc2713

[5] Vincent-Salomon A, Raynal V, Lucchesi C, Gruel N, Delattre O (2008) ESR1 gene amplification in breast cancer: a common phenomenon? Nat Genet 40: 809; author reply 810-802 doi:10.1038/ng0708-809b. PubMed: 1858396718583966. 10.1038/ng0708-809a 18583967

[6] Tomita S, Zhang Z, Nakano M, Ibusuki M, Kawazoe T et al. (2009) Estrogen receptor alpha gene ESR1 amplification may predict endocrine therapy responsiveness in breast cancer patients. Cancer Sci 100: 1012-1017. doi:10.1111/j.1349-7006.2009.01145.x. PubMed: 19320640. 19320640 10.1111/j.1349-7006.2009.01145.x PMC11159263

[7] Holst F, Stahl PR, Ruiz C, Hellwinkel O, Jehan Z et al. (2007) Estrogen receptor alpha (ESR1) gene amplification is frequent in breast cancer. Nat Genet 39: 655-660. doi:10.1038/ng2006. PubMed: 17417639. 17417639 10.1038/ng2006

[8] Nielsen KV, Ejlertsen B, Müller S, Møller S, Rasmussen BB et al. (2011) Amplification of ESR1 may predict resistance to adjuvant tamoxifen in postmenopausal patients with hormone receptor positive breast cancer. Breast Cancer Res Treat 127: 345-355. doi:10.1007/s10549-010-0984-y. PubMed: 20556506. 20556506 10.1007/s10549-010-0984-y

[9] Ejlertsen B, Aldridge J, Nielsen KV, Regan MM, Henriksen KL et al. (2012) Prognostic and predictive role of ESR1 status for postmenopausal patients with endocrine-responsive early breast cancer in the Danish cohort of the BIG 1-98 trial. Annals Oncology Off J Eur Soc Medical Oncology/ESMO 23: 1138-1144. doi:10.1093/annonc/mdr438. PubMed: 21986093. 10.1093/annonc/mdr438 PMC3335246 21986093

[10] Cai Q, Shu XO, Jin F, Dai Q, Wen W et al. (2003) Genetic polymorphisms in the estrogen receptor alpha gene and risk of breast cancer: results from the Shanghai Breast Cancer Study. Cancer epidemiology, biomarkers & prevention : a publication of the American Association for Cancer Research, cosponsored by the American Society of Preventive Oncology 12: 853-859 14504194

[11] Hill SM, Fuqua SA, Chamness GC, Greene GL, McGuire WL (1989) Estrogen receptor expression in human breast cancer associated with an estrogen receptor gene restriction fragment length polymorphism. Cancer Res 49: 145-148. PubMed: 2562795. 2562795

[12] Crewe HK, Notley LM, Wunsch RM, Lennard MS, Gillam EM (2002) Metabolism of tamoxifen by recombinant human cytochrome P450 enzymes: formation of the 4-hydroxy, 4'-hydroxy and N-desmethyl metabolites and isomerization of trans-4-hydroxytamoxifen. Drug Metab Dispos Biol Fate Chem 30: 869-874. doi:10.1124/dmd.30.8.869. PubMed: 12124303. 12124303 10.1124/dmd.30.8.869

[13] Desta Z, Zhao X, Shin JG, Flockhart DA (2002) Clinical significance of the cytochrome P450 2C19 genetic polymorphism. Clin Pharmacokinet 41: 913-958. doi:10.2165/00003088-200241120-00002. PubMed: 12222994. 12222994 10.2165/00003088-200241120-00002

[14] Nishiyama T, Ogura K, Nakano H, Ohnuma T, Kaku T et al. (2002) Reverse geometrical selectivity in glucuronidation and sulfation of cis- and trans-4-hydroxytamoxifens by human liver UDP-glucuronosyltransferases and sulfotransferases. Biochem Pharmacol 63: 1817-1830. doi:10.1016/S0006-2952(02)00994-2. PubMed: 12034366. 12034366 10.1016/s0006-2952(02)00994-2

[15] Lévesque E, Beaulieu M, Green MD, Tephly TR, Bélanger A et al. (1997) Isolation and characterization of UGT2B15(Y85): a UDP-glucuronosyltransferase encoded by a polymorphic gene. Pharmacogenetics 7: 317-325. doi:10.1097/00008571-199708000-00007. PubMed: 9295060. 9295060 10.1097/00008571-199708000-00007

[16] Dezentje VO, Schaik RHV, Vletter-Bogaartz JM, Wessels JA, Hille ET et al. (2010) Pharmacogenetics of tamoxifen in relation to disease-free survival in a Dutch cohort of the tamoxifen exemestane adjuvant multinational (TEAM) trial. 2010 ASCO, Annual Meeting.

[17] Ruiter R, Bijl MJ, van Schaik RH, Berns EM, Hofman A et al. (2010) CYP2C19*2 polymorphism is associated with increased survival in breast cancer patients using tamoxifen. Pharmacogenomics 11: 1367-1375. doi:10.2217/pgs.10.112. PubMed: 21047200. 21047200 10.2217/pgs.10.112

[18] Nowell SA, Ahn JY, Rae JM, Scheys JO, Trovato A et al. (2005) Association of genetic variation in tamoxifen-metabolizing enzymes with overall survival and recurrence of disease in breast cancer patients. Breast Cancer Res Treat 91: 249-258. doi:10.1007/s10549-004-7751-x. PubMed: 15952058. 15952058 10.1007/s10549-004-7751-x

[19] Goldhirsch A, Wood WC, Coates AS, Gelber RD, Thürlimann B et al. (2011) Strategies for subtypes--dealing with the diversity of breast cancer: highlights of the St. Gallen International Expert Consensus on the Primary Therapy of Early Breast Cancer 2011. Ann Oncol 22: 1736-1747. doi:10.1093/annonc/mdr304. PubMed: 21709140. 21709140 10.1093/annonc/mdr304 PMC3144634

[20] Zaczek A, Markiewicz A, Jaskiewicz J, Pienkowski T, Rhone P et al. (2010) Development and Evaluation of Clinical Significance of a Real-Time PCR Assay for TOP2A Copy Number Determination in Breast Cancer. Ann Oncol 21: 57-57.

[21] Hellemans J, Mortier G, De Paepe A, Speleman F, Vandesompele J (2007) qBase relative quantification framework and software for management and automated analysis of real-time quantitative PCR data. Genome Biol 8: R19-. PubMed: 17291332. 17291332 10.1186/gb-2007-8-2-r19 PMC1852402

[22] Yaich L, Dupont WD, Cavener DR, Parl FF (1992) Analysis of the PvuII restriction fragment-length polymorphism and exon structure of the estrogen receptor gene in breast cancer and peripheral blood. Cancer Res 52: 77-83. PubMed: 1345763. 1345763

[23] de Morais SM, Wilkinson GR, Blaisdell J, Nakamura K, Meyer UA et al. (1994) The major genetic defect responsible for the polymorphism of S-mephenytoin metabolism in humans. J Biol Chem 269: 15419-15422. PubMed: 8195181. 8195181

[24] Ebeshi B, Bolaji O, Masimirembwa C (2011) Allele and genotype frequencies of cytochrome P450 2B6 and 2C19 genetic polymorphisms in the Nigerian populations: Possible implication on anti-retroviral and anti-malarial therapy. Int J Med Medical Sciences 3: 193-200.

[25] McShane LM, Altman DG, Sauerbrei W, Taube SE, Gion M et al. (2005) REporting recommendations for tumour MARKer prognostic studies (REMARK). Br J Cancer 93: 387-391. doi:10.1038/sj.bjc.6602678. PubMed: 16106245. 16106245 10.1038/sj.bjc.6602678 PMC2361579

[26] Reis-Filho JS, Drury S, Lambros MB, Marchio C, Johnson N et al. (2008) ESR1 gene amplification in breast cancer: a common phenomenon? Nat Genet 40: 809-810; author reply 810-802 doi:10.1038/ng0708-809b. PubMed: 1858396718583966. 10.1038/ng0708-809b 18583966

[27] Ooi A, Inokuchi M, Harada S, Inazawa J, Tajiri R et al. (2012) Gene amplification of ESR1 in breast cancers--fact or fiction? A fluorescence in situ hybridization and multiplex ligation-dependent probe amplification study. J Pathol 227: 8-16. doi:10.1002/path.4064. PubMed: 22170254. 22170254 10.1002/path.3974

[28] Moelans CB, Monsuur HN, de Pinth JH, Radersma RD, de Weger RA et al. (2010) ESR1 amplification is rare in breast cancer and is associated with high grade and high proliferation: a multiplex ligation-dependent probe amplification study. Anal Cell Pathol 33: 13-18. PubMed: 215417332042168820966540. 10.3233/ACP-CLO-2010-0527 PMC4605667 20966540

[29] Brown LA, Hoog J, Chin SF, Tao Y, Zayed AA et al. (2008) ESR1 gene amplification in breast cancer: a common phenomenon? Nat Genet 40: 806-807; author reply 810-802 doi:10.1038/ng0708-806. PubMed: 18583964. 18583964 10.1038/ng0708-806 PMC2846830

[30] Reynisson E, Josefsen MH, Krause A, Hoorfar J (2006) Evaluation of probe chemistries and platforms to improve the detection limit of real-time PCR. J Microbiol Methods 66: 206-216. doi:10.1016/j.mimet.2005.11.006. PubMed: 16364478. 16364478 10.1016/j.mimet.2005.11.006

[31] Albertson DG (2012) ESR1 amplification in breast cancer: controversy resolved? J Pathol 227: 1-3. doi:10.1002/path.3999. PubMed: 22322671. 22322671 10.1002/path.3999

[32] Lin CH, Liu JM, Lu YS, Lan C, Lee WC et al. (2013) Clinical significance of ESR1 gene copy number changes in breast cancer as measured by fluorescence in situ hybridisation. J Clin Pathol, 66: 140–5. PubMed: 23268322. 23268322 10.1136/jclinpath-2012-200929

[33] Horlings HM, Bergamaschi A, Nordgard SH, Kim YH, Han W et al. (2008) ESR1 gene amplification in breast cancer: a common phenomenon? Nat Genet 40: 807-808. doi:10.1038/ng0708-807. PubMed: 18583965. 18583965 10.1038/ng0708-807

[34] Ryan J, Canonico M, Carcaillon L, Carrière I, Scali J et al. (2012) Hormone treatment, estrogen receptor polymorphisms and mortality: a prospective cohort study. PLOS ONE 7: e34112. doi:10.1371/journal.pone.0034112. PubMed: 22457817. 22457817 10.1371/journal.pone.0034112 PMC3311587

[35] Schroth W, Antoniadou L, Fritz P, Schwab M, Muerdter T et al. (2007) Breast cancer treatment outcome with adjuvant tamoxifen relative to patient CYP2D6 and CYP2C19 genotypes. J Clin Oncol 25: 5187-5193. doi:10.1200/JCO.2007.12.2705. PubMed: 18024866. 18024866 10.1200/JCO.2007.12.2705

[36] Okishiro M, Taguchi T, Kim SJ, Shimazu K, Tamaki Y et al. (2009) Genetic Polymorphisms of CYP2D6*10 and CYP2C19* 2,*3 Are not Associated With Prognosis, Endometrial Thickness, or Bone Mineral Density in Japanese Breast Cancer Patients Treated With Adjuvant Tamoxifen. Cancer 115: 952-961. doi:10.1002/cncr.24111. PubMed: 19156902. 19156902 10.1002/cncr.24111

[37] Altshuler DL, Durbin RM, Abecasis GR, Bentley DR, Chakravarti A et al. (2010) A map of human genome variation from population-scale sequencing. Nature 467: 1061-1073. doi:10.1038/nature09534. PubMed: 20981092. 20981092 10.1038/nature09534 PMC3042601

