# Supplementary material for: Prognostic Significance of ESR1 Amplification and ESR1 PvuII, CYP2C19*2, UGT2B15*2 Polymorphisms in Breast Cancer Patients
Source: PLoS One. 2013 Aug 8;8(8):e72219. doi: 10.1371/journal.pone.0072219 (PMC3738574; doi:10.1371/journal.pone.0072219)
Supplement: Text S1 — Description of ER, PgR, Ki-67, HER2 status determination in formalin fixed paraffin embedded tumor samples. (PDF) [file pone.0072219.s001.pdf]

Text S1

ER, PgR, Ki-67 and HER2 status determination in formalin fixed paraffin embedded tumor samples

A mouse monoclonal antibodies against estrogen receptor alpha (ER α, clone 1D5, Dako, Denmark), progesterone receptor (PgR, clone 636, Dako, Denmark) and Ki-67 (Ki-67, clone MIB1, Dako, Denmark) were used, according to the manufacturer's instructions, at a dilution of 1:50 (ER, PgR) or 1:600 (Ki67) and antigen retrieval was performed at neutral pH by water-bath heating at 90 °C for 30 min. Envision Dako system was used for visualization in case of ER and PgR staining and Novolink Polymer Detection System (Novocastra, Germany) for Ki-67 visualization. Samples were examined locally. For ER and PgR evaluation of the immunohistochemical nuclear staining was performed based on Allred score [1] or immunoreactivity of any intensity in at least 10% of the tumor cells (for older tumor samples). Ki-67 positive nuclear staining above the background was scored as a percentage of positive cells [2]. HER2 receptor status was determined with the use of Hercept Test Kit (Dako, Denmark) according to the manufacturer's guidelines. Positive result was considered as 3+ score. The 2+ score as equivocal was tested for HER2 gene amplification by fluorescence in situ hybridization (FISH) with PathVision HER2 DNA Probe Kit (Abbott Molecular, USA), according to the manufacturer's instructions. The mean numbers of HER2 and centromer 17 signals were estimated for each tumor sample. A ratio of HER2/CEP-17 ≥2 was considered as HER2 amplification and HER2-positive result.

1. Allred DC, Harvey JM, Berardo M, Clark GM: Prognostic and predictive factors in breast cancer by immunohistochemical analysis. Mod Pathol 1998, 11(2):155-168.
2. Cheang MCU, Chia SK, Voduc D, Gao DX, Leung S, Snider J, Watson M, Davies S, Bernard PS, Parker JS et al: Ki67 Index, HER2 Status, and Prognosis of Patients With Luminal B Breast Cancer. J Natl Cancer I 2009, 101(10):736-750.
