# Supplementary material for: Prognostic Significance of ESR1 Amplification and ESR1 PvuII, CYP2C19*2, UGT2B15*2 Polymorphisms in Breast Cancer Patients
Source: PLoS One. 2013 Aug 8;8(8):e72219. doi: 10.1371/journal.pone.0072219 (PMC3738574; doi:10.1371/journal.pone.0072219)
Supplement: Figure S1 — ESR1 gene dosage in breast cancer samples according to ER status measured by IHC. (PDF) [file pone.0072219.s002.pdf]

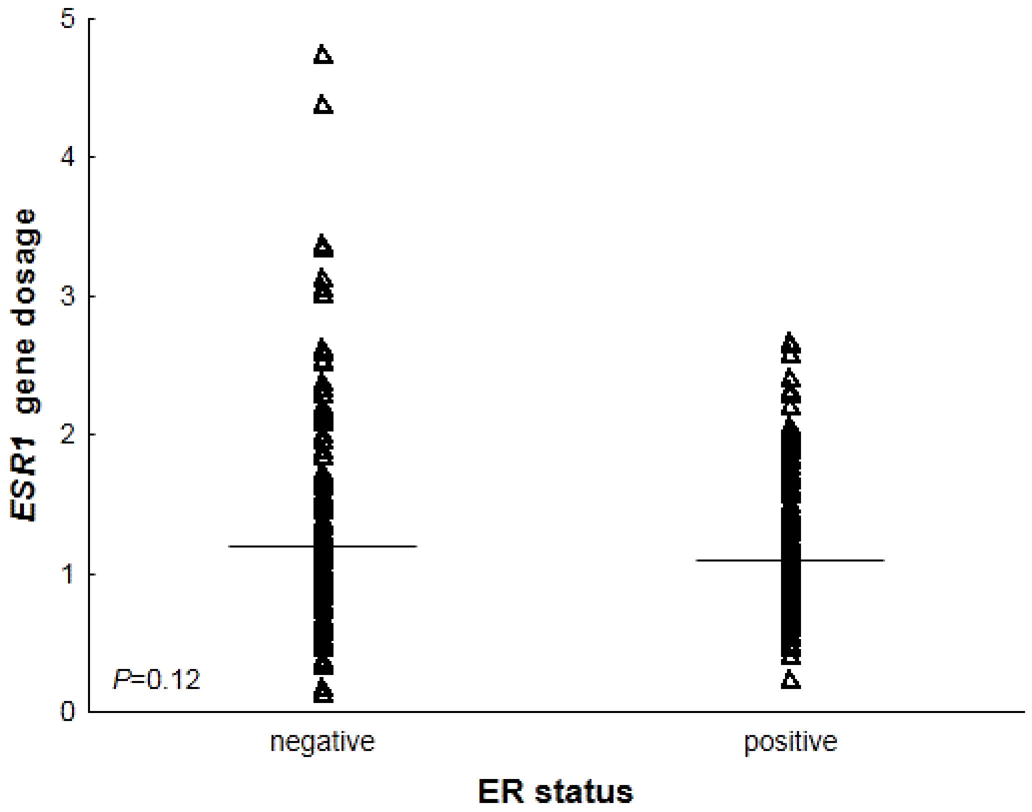

Figure S1. *ESR1* gene dosage in breast cancer samples according to ER status measured by IHC. Horizontal line indicates median value of *ESR1* gene dosage.
