# Supplementary material for: Prognostic Significance of ESR1 Amplification and ESR1 PvuII, CYP2C19*2, UGT2B15*2 Polymorphisms in Breast Cancer Patients
Source: PLoS One. 2013 Aug 8;8(8):e72219. doi: 10.1371/journal.pone.0072219 (PMC3738574; doi:10.1371/journal.pone.0072219)
Supplement: Table S1 — Univariate and multivariate analysis for DFS and OS according to molecular markers and clinicopathological variables. (PDF) [file pone.0072219.s003.pdf]

Table S1. Univariate and multivariate analysis for DFS and OS according to molecular markers and clinicopathological variables.

**Univariate analysis**	DFS		OS	
**Variable**	***P***	**Hazard ratio (95% CI)**	***P***	**Hazard ratio (95% CI)**
Menopausal status (>50 vs. ≤ 50 yr.)	0.68	0.68 (0.68-0.68)	**0.03**	2.90 (1.09-7.14)
Tumor size (T1-2 vs. T3-4)	**<0.00001**	4.38 (2.68-7.15)	**<0.00001**	4.42 (2.33-8.37)
Lymph nodes (negative vs. positive)	**<0.00001**	3.32 (2.02-5.46)	**0.0009**	3.00 (1.57-5.75)
ER (negative vs. positive)	0.33	0.79 (0.50-1.26)	0.75	0.91 (0.49-1.68)
PgR (negative vs. positive)	**0.01**	0.55 (0.34-0.88)	0.20	0.67 (0.36-1.23)
Histological type	0.20	0.65 (0.34-1.25)	0.16	0.51 (0.20-1.30)
HER2 (normal vs. increased)	**0.02**	2.27 (1.15-4.51)	0.57	1.32 (0.50-3.48)
Tumor size (>2cm vs. ≤ 2cm)	**0.005**	2.12 (1.26-3.57)	**0.07**	1.84 (0.95-3.56)
G (1-2 vs.3)	**0.02**	1.97 (1.12-3.46)	**0.06**	2.03 (0.97-4.27)
ESR1 gene dosage (normal vs. increased)	**0.0006**	1.68 (1.25-2.26)	**0.02**	1.61 (1.07-2.41)
**Multivariate analysis**	DFS		OS	
**Variable**	***P***	**Hazard ratio (95% CI)**	***P***	**Hazard ratio (95% CI)**
Menopausal status (>50 vs. ≤ 50 yr.)	Not included		**0.01**	12.92 (1.74-96.14)
Tumor size (T1-2 vs. T3-4)	0.15	2.99 (0.68-13.23)	**0.04**	2.99 (1.06-8.40)
Lymph nodes (negative vs. positive)	**0.01**	3.28 (1.30-8.27)	0.11	2.05 (0.86-4.90)
PgR (negative vs. positive)	0.41	0.69 (0.28-1.67)	Not included	
G (1-2 vs.3)	0.99	1.00 (0.39-2.56)	0.20	1.65 (0.77-3.54)
HER2 (normal vs. increased)	**0.03**	3.14 (1.12-8.80)	Not included	
ESR1 gene dosage (normal vs. increased)	0.11	1.54 (0.92-2.59)	0.46	1.22 (0.72-2.06)

Abbreviations: OS – overall survival, DFS – disease free survival, 95% CI – 95% confidence interval, N – number of cases. Significant *P* values are given in bold.
